# Updates on the Pathophysiology and Therapeutic Potential of Extracellular Vesicles with Focus on Exosomes in Rheumatoid Arthritis

**DOI:** 10.2147/JIR.S465653

**Published:** 2024-07-19

**Authors:** Ioulia Karolina Gavriilidi, Joanna Wielińska, Katarzyna Bogunia-Kubik

**Affiliations:** 1 Laboratory of Clinical Immunogenetics and Pharmacogenetics, Hirszfeld Institute of Immunology and Experimental Therapy, Polish Academy of Sciences, Wrocław, Poland

**Keywords:** extracellular vesicles, exosomes, rheumatoid arthritis, therapy, microRNA

## Abstract

Rheumatoid arthritis (RA) is an incurable autoimmune disease with high morbidity and socioeconomic burden. Advances in therapeutics have improved patients’ quality of life, however due to the complex disease pathophysiology and heterogeneity, 30% of patients do not respond to treatment. Understanding how different genetic and environmental factors contribute to disease initiation and development as well as uncovering the interactions of immune components is key to the implementation of effective and safe therapies. Recently, the role of extracellular vesicles (EVs) in RA development and possible treatment has been an area of interest. EVs are small lipid-bound entities, often containing genetic material, proteins, lipids and amino acids, facilitating paracrine intercellular communication. They are secreted by all cells, and it is believed that they possess regulatory functions due to high complexity and functional diversity. Although it has been shown that EVs participate in RA pathophysiology, through immune modulation, their exact role remains elusive. Furthermore, EVs could be a promising therapeutic agent in various diseases including RA, due to their biocompatibility, low toxicity and possible manipulation, but further research is required in this area. This review provides a comprehensive discussion of disease pathophysiology and summarizes the latest knowledge regarding the role and therapeutic potential of EVs in RA.

## Introduction

Rheumatoid arthritis (RA) is a chronic autoimmune disease characterized by widespread inflammation, which manifests primarily in the synovial joints but can also affect extra-articular organs within the body, such as the heart, the liver, the nervous system and the lungs. Clinical presentation includes swollen joints, stiffness, pain and deformations, often presented in a symmetrical pattern. Landmark of the disease is synovial inflammation. If untreated, RA can lead to loss of joint function, disability and complications due to systemic inflammation, such as stroke, heart disease or cognitive decline. Currently, the disease is incurable with high morbidity and approximately 30% of patients not responding to any type of treatment.^1^,^2^

RA has been one of the most prevalent chronic inflammatory illnesses worldwide, with 18 million people living with the disease in 2019, according to the World Health Organization. The RA development varies between geographic locations from 0.25% to 1% which is attributed to environmental and genetic factors. Increased incidence has been observed in industrialized countries, possibly due to higher environmental exposure to tobacco and pollution. Risk factors include sex and age with women above the age of 55 being mostly affected and up to 70% of all patients being women.^3^ In Poland, the prevalence in 2019 was 0.9% which accounts for approximately 400,000 patients. The occurrence of the disease has been increasing historically, however thanks to medicinal and pharmacological advances the morbidity is decreasing.^4^

The development of RA is a complex pathophysiological process which is not yet fully understood. Abnormalities in the functionality of the immune system lead to the generation of autoantibodies, anti-modified protein antibodies (AMPA); the presence of T and B-lymphocytes in the synovium; as well as hyperactivation of both the innate and adaptive immunity with various cells such as monocytes, macrophages and lymphocytes as well as cytokines such as tumor necrosis factor (TNF-α), interferon-gamma (IFN-γ), interleukin-6 (IL-6), and granulocyte-monocyte colony-stimulating factor (GM-CSF), taking part in inflammatory processes within the joints.^2^

The current first-line treatment for RA includes the implementation of disease-modifying anti-rheumatic drugs (DMARDs) and glucocorticoids, aiming for disease remission or low activity if remission cannot be achieved. DMARDs can be either synthetic or biological. Synthetics are preferably used first and include methotrexate, hydroxychloroquine, sulfasalazine, azathioprine, cyclosporine and leflunomide for inflammation suppression. Biological DMARDs include anti-TNF-α inhibitors such as etanercept, infliximab, adalimumab, golimumab, and certolizumab pegol; IL-6 receptor inhibitors mainly, tocilizumab and sarilumab; T-cell co-stimulation inhibitors, abatacept (CTLA4-Ig), and the anti-CD20 B-cell depleting monoclonal antibody such as rituximab. Some other synthetic DMARDs with targeted mechanisms of action include the Janus kinases (JAK) inhibitors such as tofacitinib, baricitinib, and upadacitinib. Glucocorticoids are used short term for the management of flares or as combination therapy with DMARDs. Low doses can be used daily for disease control. Finally, non-steroidal anti-inflammatory drugs (NSAIDs) are often incorporated for pain and inflammation relief. Those do not have any particular effect on underlying disease-driving mechanisms.^5–7^

Novel therapeutic approaches such as gene therapy, mesenchymal stem cell (MSC) therapy or targeting regulatory miRNAs are currently under investigation.^8–10^ One interesting and perhaps novel approach includes the use of extracellular vesicles (EVs) as possible therapeutic agents. Notably, EVs have been shown to participate in the pathophysiology of RA and may constitute an important factor in disease development and progression.^10–12^

Extracellular vesicles are lipid membrane-bound entities, secreted by all cells into the extracellular space; they can be found in all bodily fluids and participate in various physiological and pathological processes. Their cargo includes nucleic acids, lipids and proteins, and their main role is to facilitate intercellular communication. Three types of EVs have been characterized; microvesicles, exosomes and apoptotic bodies. The latest are released during apoptosis and often contain intact organelles. Microvesicles are formed by direct outward budding or pinching and are the largest of EVs, with a diameter of 100nm to 1 μm. Exosomes are EVs of interest when it comes to pathophysiological, therapeutic and prognostic research in autoimmune diseases and cancer. They are formed by endosomal route and their size varies from 20 to 200 nm.^13^,^14^ What makes them particularly interesting is their high complexity and functional diversity which suggest important regulatory roles. The aim of this review is to present the latest insights into the pathophysiology of rheumatoid arthritis and to explore the pathological and therapeutic potential of EVs, specifically exosomes.

## Pathophysiology of Rheumatoid Arthritis

The interplay of multiple factors including, genetic predisposition and environmental exposure (ie tobacco smoke, pollution, dust, silica, microbiome, pathogens) results in the abnormal function of the immune system in RA. On the genetic side, more than 150 genes have been associated with RA initiation and development. As in other autoimmune disorders, a strong association with the disease has been identified with the major histocompatibility complex (MHC) class II alleles, specifically of the *HLA-DRB1* locus, coding for a conserved domain of 5 amino acids (70–74 of the HLA-DRβ chain), named the “shared epitope” (originally: P4 peptide-binding pocket), that exhibits increased hypervariability (alleles: HLA-DRB1*04, HLA-DRB1*01, and HLA-DRB1*10).^15^ Other RA associated genes, which exhibit polymorphism are *PAD14, PTPN22, CTLA4, IL-2RA, STAT4, TRAF1, CCR6, and IRF5,* and in severe RA cases, single nucleotide polymorphism, in *PSORS1C1, PTPN22, and MIR146A*, has been observed. The hereditability of RA counts approximately 60%.^16^,^17^

**Figure 1:**
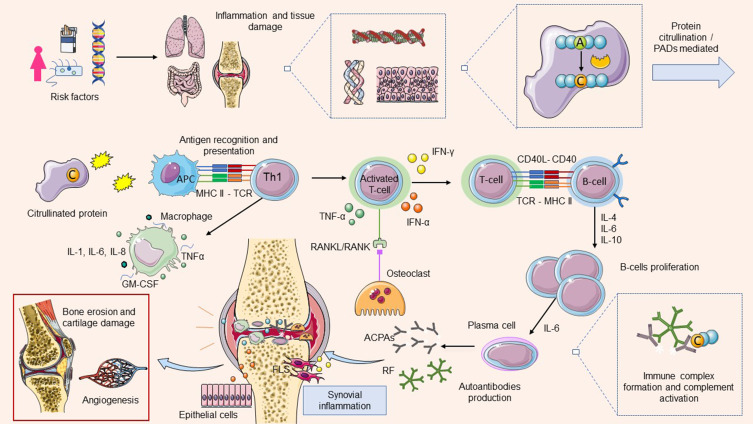
The pathophysiology of rheumatoid arthritis (RA). Multiple risk factors contribute to disease initiation which manifests through inflammation within tissues. Tissue inflammation contributes to the expressions of peptidyl arginine deiminase (PAD) which participates in the process of protein citrullination. Citrullinated proteins are detected by antigen-presenting cells (APC) and activation of adaptive and innate immunity occurs with the production of pro-inflammatory cytokines and chemokines. Over activation of immune response leads to the generation of autoantibodies by plasma cells in the synovium, the formation of immune complexes and activation of the complement system. The cytokines produced by macrophages and T-cells, stimulate synovial cells to proliferate, resulting in the generation of pannus and consequently bone erosion and damage to the articular cartilage. Abnormal activation and differentiation of osteoclast, which is a T-cell and macrophage-mediated process regulated by granulocyte-macrophage colony stimulating factor (GM-CSF) stand the nuclear factor κB receptor activator ligand (RANKL), induce bone resorption in RA. Repeated activation of the immune response, results in the generation of anti-citrullinated protein antibodies (ACPAs), produced by B-cells, which recognize citrullinated peptides.

A well-documented example of RA development, is the interplay between tobacco and genetic predisposition (Figure 1). In particular, cigarette smoking induces inflammation and tissue damage at the site of lungs as well as the expression of peptidyl arginine deiminase (PAD). This, in turn, favors the formation of citrullinated proteins through post-transcriptional modification, where arginine is substituted by citrulline in the presence of PADs. The shared epitope HLA-DRB1, presents the citrullinated peptide epitopes to the adaptive immune system, specifically T helper 1 cells (Th1), via antigen-presenting cells (APC), ie dendritic cells. This triggers T-cell activation through the release of IL-2 and macrophage activation by IL-17 and IFN-γ. Activated T-cells release additional IFN-γ and α, while B-cells can also act as antigen-presenting cells for T-cells through MHC Class I. In addition, Th1 cells stimulate B-cells to proliferate and differentiate into plasma cells via the action of IL-6. Eventually, autoantibodies start being produced by plasma cells in the synovium and the formation of immune complexes and activation of the complement system occur. During synovial inflammation, activated macrophages secrete IL-1, IL-6, IL-8, TNF, GM-CSF, prostaglandins, leukotrienes and nitric oxide. The cytokines produced by macrophages and T-cells stimulate synovial cells to proliferate, resulting in the generation of pannus which is the hyperplastic and swollen synovial membrane composed of fibroblasts, myofibroblasts and inflammatory cells. Pannus formation is irreversible and over time leads to bone erosion and damage to the articular cartilage of the joint. Endothelial cells of the synovium are also triggered by the presence of cytokines and chemokines and attract more immune cells enhancing, thus, inflammation. Another aspect of the pathophysiology of RA includes the fibroblast-like synovial cells (FLSs), which under normal conditions produce components of the synovium and cartilage, such as lubricin and hyaluronic acid. However, in RA, they exhibit an IL-17-mediated aggressive phenotype and produce metalloproteinases (MMPs) such as MMP1, MMP2, MMP3 and MMP10 which are able to digest proteins of the articular cartilage.^16^,^18^,^19^ Additionally, they produce pro-inflammatory cytokines such as IL-6 and GM-CSF, which further induce inflammation.^19^

In RA, inflammatory cytokines lead to abnormal activation and differentiation of osteoclast, a process which has been shown to be T-cell mediated. Notably, macrophages and monocytes are osteoclast precursors, and osteoclast differentiation is mainly regulated by GM-CSF and the nuclear factor κB receptor activator ligand (RANKL). The first is produced by macrophages infiltrating the synovium, while the second is expressed by activated T-cells, osteoblasts and FLSs. RANKL is overexpressed during inflammation and is capable of binding RANK receptor on the surface of osteoclasts to induce osteoclastogenesis and consequently, bone resorption.^20^,^21^ Involved in this process is also the JAK/STAT signaling pathway. Specifically, the JAK/STAT pathway is activated by pro-inflammatory cytokines such as IL-6 and mediates RANKL expression in osteoblasts as well as osteoclast maturation. Thus, JAK/STAT has been shown to be a bone destruction mediator. As a result of chronic inflammation and hyperplasia, angiogenesis occurs, favouring the migration of additional inflammatory cells to the joints. The inhibitory effect on the RANKL/RANK binding has osteoprotegerin (OPG), and an imbalance of RANKL and OPG has been observed in RA.^22^,^23^

Repeated activation of the immune response, results in the generation of anti-citrullinated protein antibodies (ACPAs), produced by B-cells, which recognize citrullinated peptides in proteins such as vimentin, fibronectin, fibrinogen, histones, and type 2 collagen. Also, characteristic of RA is the presence of the rheumatoid factor (RF) which is usually an IgM type of antibody (can be IgA or IgE also) binding to the Fc domain of IgG antibodies. Both ACPAs and RF are characteristic and prognostic markers for RA and can be detected years before the clinical manifestation of the disease. To clarify, ACPAs, are frequently present in RA patients, carrying the shared epitope of the HLA-DRB1 alleles. Those who are seronegative for ACPAs, have a predisposition for other inflammatory diseases, for example, seronegative RA or psoriatic arthritis.^20^

## Epigenetics in Rheumatoid Arthritis

Although generic models based on genetic heterogeneity explain various aspects of RA pathophysiology, epigenetic mechanisms are also implicated in disease development, progression and response to treatment.

Micro RNAs (miRNAs) are short non-coding mRNA sequences of 21–23 nucleotide length, participating in gene silencing, mRNA degradation and translation regulation. Many studies demonstrate their implications in a wide range of diseases, including autoimmune, degenerative and cancer as well as their ability to regulate signaling pathways and immune responses. Their altered expression has been linked to RA pathophysiology, specifically, hyperplasia, bone damage and inflammation (Table 1).^24^ One of the most studied miRNAs is miR-146a which is characteristically overexpressed in synovial T-cells and positively correlated with disease activity.^25^,^26^ The low-expression miRNAs was found to be regulatory for 17 pathways associated with TNF and chemokine signaling, suggesting that low expression of miRNA has a regulatory role that contributes to the disease.^27^ Two miRNAs linked to bone destruction are miR-145-5p and miR-106b both implicated in the RANK/RANKL/OPG pathway. Their overexpression has been shown to increase osteoclast differentiation and bone degradation. The lower expression of miR-145-5p and miR-34a-3p is associated with the regulation of proliferation of FLSs, apoptosis and expression of proinflammatory cytokines such as TNF-α and IL-6. The first works by targeting STAT3, while the second arrests the cell cycle in the G1 phase when increased; consequently, none of these actions happen in RA when both miRNAs are decreased.^28^,^29^ Although miRNAs have the potential as biomarkers or therapeutic mediators, their main disadvantage is lack of specificity. Their sequences are non-specific and can target multiple genes to alter their expression, while often their presence is not associated with RA exclusively, but with other conditions as well.^30^

**Table 1:** MicroRNA Functions in RA Pathophysiology

miRNA	Function	Cell Derived	Exosome Derived	References
miR-146a	Inhibition of T-cell apoptosis, Tregs/Th imbalance, FLSs proliferation	x	x	[31–33]
miR-145-5p	Increase osteoblast differentiation and bone degradation	x		[28,34]
miR-106b	Increase osteoblast differentiation and bone degradation	x	x	[35,36]
miR-92a	Proliferation and migration of FLSs/apoptosis resistance	x	x	[37,38]
miR-335p	Cartilage repair		x	[10]
miR-438-5p	Cartilage repair		x	[10]
miR-17	Suppression of TNF-α signaling/Inhibition of Tregs	x	x	[39,40]
miR-140-5p	Regulation of FLSs proliferation, apoptosis and expression of TNF-α and IL-6.	x		[41]
miR-34a-3p	Regulation of FLSs proliferation and expression of TNF-α, IL-6 and MMPs.	x		[42]
miR-let-7b	Remodeling anti-inflammatory macrophages into M1		x	[11]
miR-548a-3p	Regulation of inflammatory response		x	[43]
miR-23a-3p	Cartilage repair		x	[44]
miR-27a-3p	Proliferation, apoptosis, secretion of TNF-α, IL-1, IL-6 and IL-8	x	x	[45,46]
miR-221-3p	Bone Signaling / Inhibition of osteoclast differentiation	x	x	[47]

## Extracellular Vesicles and Exosomes in the Pathophysiology of RA

Extracellular vesicles are secreted by almost all cells in the body and facilitate intercellular communication both during physiological processes and pathological conditions. They do so by, cell-to-cell contact, receptor-ligand binding, release of content into the extracellular space, endocytosis and fusion with the target cell membrane which ultimately aims, for EV cargo release within the cell.^48^ Through cargo release, EVs can alter the biochemistry of their target and modify gene expression. Importantly, during immunological response, cells of the immune system are able to communicate with one another through EV secretion. This cell-to-cell communication facilitates the exchange of information and cellular cross-talk between innate and adaptive immunity which can initiate or further enhance inflammatory response in diseases such as RA.^49^,^50^

**Figure 2:**
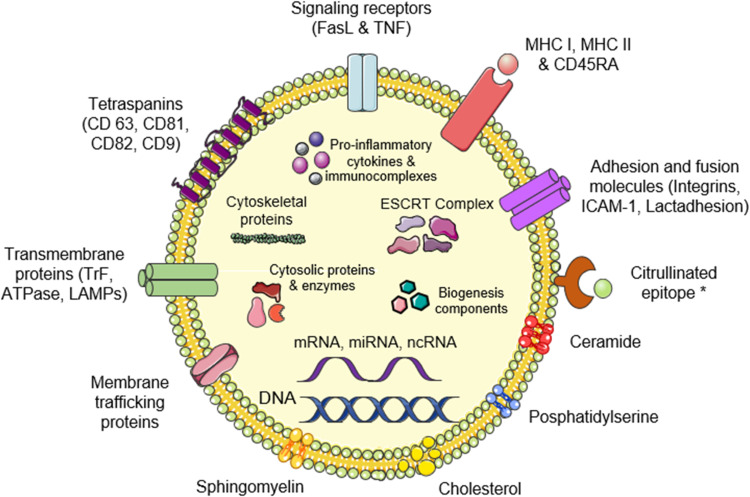
Extracellular vesicle structure and cargo. The contents of EVs depend on their biogenesis, the cell of origin, the conditions during formation and ultimately their role. In rheumatoid arthritis, citrullinated epitopes, pro-inflammatory cytokines, immune complexes and MHC class I and II molecules have been observed as part of EVs composition. *Identified in EVs of RA patients.

**Abbreviations**: FasL, Fas ligand; TNF, tumour necrosis factor; MHC, major histocompatibility complex; ICAM-1, intercellular adhesion molecule 1; LAMPs, lysosomal associated membrane proteins; TrF, Transformer proteins; ESCRT, endosomal sorting complex required for transport.

In different biological fluids, different types of EVs with variable contents can be found. For example, in cerebrospinal fluid EVs can contain neural and synaptic proteins (eg, Tau protein, amyloid precursor, synaptophysin and synaptotagmin), biomarkers for neurodegenerative disorders (eg, amyloid precursor protein, the prion protein and DJ-1), characteristic surface markers (ie, Neural Cell Adhesion Molecule) or neurotransmitters (ie, glutamate and gamma-aminobutyric acid (GABA)).^51–53^ In the synovial fluid of RA patients, the composition of EVs is rather different (Figure 2). In any case, the contents are dependent on the cell of origin, the cell conditions, internal and external stimuli and the machinery behind cargo sorting. Exosomes, in particular, are generated through the endosomal route and in the form of intraluminal vesicles. Cargo is loaded during EV biogenesis and for exosomes, it begins with lipids and membrane-associated proteins being clustered in discrete membrane microdomains at the sites of the limiting membrane of the multivesicular endosome (MVE). These domains also recruit cytosolic proteins and mRNA that will be further sorted into vesicles as inward budding and fission of small membrane vesicles containing sequestered cytosol occur. Crucial (but not necessary) in biogenesis and cargo sorting is the endosomal sorting complex required for transport (ESCRT) machinery. This machinery (ESCRT-0, ESCRT-I, ESCRT-II, ESCRT-III) in association with its accessory proteins (Alix, VPS4, VTA-1) incorporates, cargoes to the limiting membrane and recruits additional complexes that facilitate budding and fission. Independent from ESCRT sorting is also possible through sphingomyelin hydrolysis, mediated by neutral type II sphingomyelinase, which generates ceramide. Ceramides either participate in membrane subdomain generation or are metabolized to sphingosine 1-phosphate thus activating the Gi-protein-coupled sphingosine 1-phosphate receptor which is an essential sorting component. Additional proteins which participate in cargo sorting are the tetraspanins CD63, CD81, CD82 and CD9 which can induce inward budding and regulate integrins. Additional mechanisms of cargo sorting include the recruitment of cytosolic proteins along with chaperone heat shock proteins and cognate heat shock proteins.^47^,^54^

Some common protein cargoes within EVs include proteins related to the biogenesis process such as ESCRT or heat shock proteins. However, according to the stimuli by which EVs are generated and the heterogeneity of the sorting process, different proteins can be loaded through cis-acting targeting signals associated with the plasma membrane as an oligomeric complex.^44^

An additional process that has been demonstrated to be crucial in terms of cargo loading, biogenesis and secretion of EVs is autophagy which is a highly conserved process of lysosome-mediated degradation of cytosolic materials, important for cellular homeostasis. One research group found key autophagy components such as LC3, ATG16L1 and LAMP2, within endothelial-derived EVs under stress conditions. Moreover, it has been demonstrated that the ATG12‐ATG3 complex (one of the ATG complexes which regulate autophagy) interacts with Alix (an ESCRT accessory protein), which regulates endosomal function and exosome biogenesis.^45^ One theory supports that LC3 protein and its family members are responsible for LC3‐dependent EV loading and secretion (LDELS) of miRNAs which is particularly interesting as miRNAs derived from exosomes possess regulatory roles in inflammatory processes.^39^ It is important to mention that, autophagy has been also described as deregulated process in RA. Autophagy markers (belcin1, Atg5, and LC3) are increased in the synovial fluid of RA patients and correlate with RA markers (RF, CCP, CRP, ESR) and disease activity.^55^ It is believed that autophagy is antagonistic to apoptosis in RA and that it promotes FLS proliferation.^56^,^57^ Other ways in which autophagy participates in disease pathophysiology include decreased bone formation through defective autophagy in osteoblasts and increased autoantigen presentation by antigen-presenting cells which undergo autophagy to produce citrullinated peptides.^58–60^ As autophagy is implicated in the generation of autoantigens by antigen-presenting cells, exosomes are carriers of autoantigens (citrullinated proteins such as Spα receptor, fibrin α-chain fragment, fibrin β-chain, fibrinogen β-chain precursor, fibrinogen D fragment, and vimentin) a connection between autophagy-exosomes crosstalk and RA is being proposed.^61^,^62^ However, more studies are required to understand the possible biochemical interactions between autophagy, exosomes and FLS and/or immune cells.^63^

In a similar pattern to apoptosis, the process of NETosis is an additional inflammation driver responsible for autoantigen generation in RA.^64^,^65^ NETosis is a form of cell death commonly seen in neutrophils in response to pro-inflammatory stimuli. It is characterized by the formation of neutrophil extracellular traps (NETs) which consist of fibrous structures made of decondensed chromatin, and granular and cytoplasmic neutrophil proteins. In terms of EVs, it has been demonstrated that a subtype of EVs characteristic for neutrophils and neutrophil-derived ectosomes (NDEs) can modulate core neutrophil responses such as delayed apoptosis, inflammatory chemokine generation, and NET formation.^66^ In addition, NETosis can stimulate the release of EVs in which case their cargo can contain histones, peptides and signaling molecules.^67^ NETs are also a source of extracellular citrullinated autoantigens, and they have been identified in the synovial tissue in rheumatoid arthritis, thus contributing to disease pathophysiology.^68^

Exosomes have been found to have abundant miRNA content. The process of the miRNA loading depends on its sequence; hence, it is tightly regulated. The ESCRT-II subcomplex has been suggested as a complex acting similarly to the RNA binding complexes, while the miRNA-induced silencing complex (miRISC) and protein argonaute 2 (AGO2) can mediate RNA-silencing. Some additional regulating mechanisms might be mediated by the KRAS–MEK signaling pathway, major vault protein 72 and Y-box-binding protein 1.^69^ Also, cellular stress can induce miRNA enrichment in EVs as well as the presence of specific motifs such as the GAGG. Finally, it has been proposed that the 3′ end uridylation of miRNAs through which nSMase2 activity increases results in the loading of miRNA.^47^

Attention has been paid to the miRNA content of exosomes which seems to have a crucial indirect regulatory role during inflammation and in bone homeostasis (Table 1). Notably, the carried miRNAs consist of a form of intercellular communication and are proposed as diagnostic biomarkers in RA. An example will be miR-221-3p which is FLS-derived and involved in bone signaling pathways or miR-146a, a miRNA responsible for inhibition of bone destruction by osteoclasts.^31–33^ Recently, Yu e*t al*, investigated the role of two exosomal miRNAs, miR-335p and miR-483-5p. First, they established that the expression levels of those miRNAs are increased in RA patient’s peripheral blood. Then, they identified a common target for these sequences, the *SRSF4* gene which is responsible for cartilage repair. The expression of this gene was decreased suggesting a potential implication in RA pathophysiology, ie blockage of cartilage repair.^10^ Further investigation of the SRSF4 protein action is, however, required. Involved in cartilage repair is also miR-23a-3p specifically by suppression of PTEN and activation of the Akt signaling pathway in chondrocytes.^34^ One interesting recent discovery regarding the pathophysiology of RA, is the association of FLS-derived exosomal lncRNA and miRNA where the lncRNA tumor necrosis factor-associated factor 1 (TRAF1) interacts with miR-27a-3p through sponging to upregulate the expression of C-X-C motif chemokine ligand 1 in chondrocytes. The same study indicates that exosomes which originate from TNF-α stimulated FLSs in RA, can suppress the proliferation and migration of chondrocytes, thus suggesting a therapeutic potential of the exosomes.^35^ Exosomes and their miRNA have been also linked with the imbalance of T-regulatory cells (Tregs)/Th cells and Tregs homeostasis. Specifically, the upregulation of miR-17 was associated with inhibition of transforming growth factor beta receptor II (TGFBR II) expression which in turn inhibited the differentiation of Tregs in vitro.^36^ Finally, researchers identified 12 differentially expressed miRNAs in EVs of RA patients, which were upregulated and linked to the Wnt signaling pathway. They also identified miRNAs which have not been described earlier in terms of RA and specific ones to distinguish between RA and ankylosing spondylitis (AS).^37^

**Figure 3:**
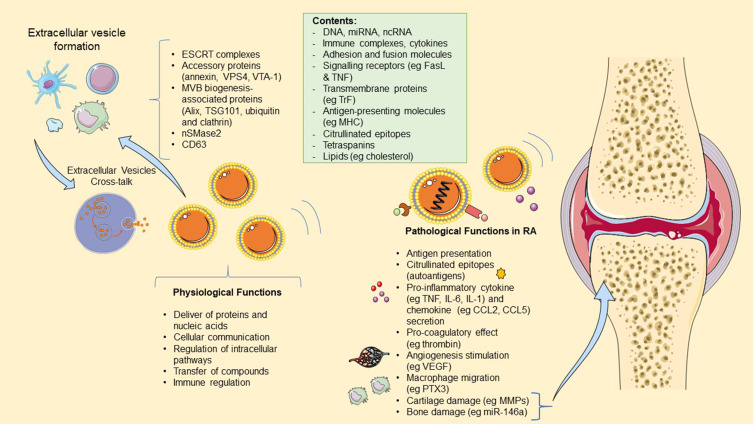
The role of extracellular vesicles (EVs) in RA. Most cells within the body can produce EVs, including the cells of the immune system. EV biogenesis requires components such as; the endosomal sorting complex required for transport (ESCRT) machinery in association with its accessory proteins (Alix, VPS4, VTA-1); II sphingomyelinase, ceramides, Gi-protein-coupled sphingosine 1-phosphate receptor, tetraspanins and integrins. They contents may vary, but in RA often includes citrullinated epitopes and antigen-presenting molecules. EV physiological functions include cellular communication and transport, although they have immunomodulatory functions such as maintaining the cross-talk between innate and adaptive immunity. In RA, EVs have pathological roles, such as antigen presentation, autoantigen function, cytokine secretion, angiogenesis and macrophage migration stimulation and pro-coagulatory effect.

**Abbreviations**: ESCRT, endosomal sorting complex required for transport; VPS4, vacuolar protein sorting 4-A; VTA-1, vacuolar protein sorting-associated 1; TSG101, tumor susceptibility gene 101; nSMase2, neutral sphingomyelinase 2; FasL, Fas ligand; TNF, tumor necrosis factor; MHC, major histocompatibility complex; Il-6, interleukin 6; IL-1, interleukin 1; CCL2, chemokine (C-C motif) ligand 2; CCL5, chemokine (C-C motif) ligand 5; VEGF, vascular endothelial growth factor; PTX3, pentraxin 3; MMPs, Metalloproteinases.

Immunomodulatory functions and crosstalk between EVs and the immune system have been described in the disease context (Figure 3). Increased levels of EVs derived from immune cells, osteoclast, FLSs and platelets have been identified in synovial fluid of RA patients, while some of the EVs, ie those derived from platelets, are also found increased in peripheral blood. The composition of EVs consists of various proteins that facilitate adhesion and fusion, glycoproteins and lipids, but importantly they can also possess antigen-presenting molecules on their surface (MHC class I and II), signaling receptors such as TNF and FasL, miRNAs and pro-inflammatory cytokines. Several studies have explored their roles both in vitro and in vivo and described possible direct and indirect mechanisms of EVs exerting pro-inflammatory and pro-coagulatory effects, promoting FLSs stimulation and angiogenesis and finally consisting a source of autoantigens themselves.^67^ The pathophysiological roles of EVs derived from cells of the immune system in RA are summarized in Table 2.

**Table 2:** Direct Pathophysiological Roles of Extracellular Vesicles in Rheumatoid Arthritis

Origin	Effect	Mediator(s)	Reference
Monocyte and T-cell -derived	Pro-inflammatory	COX-2 and m-PGES	[57]
Platelet-derived	Migration and invasion of FLSs	CXCR2-mediated signaling pathway and NF-κB	[58]
Platelet-derived	Pro-inflammatory / Stimulation of FLSs cytokine release	IL-1 receptor	[60]
Platelet-derived	Angiogenesis	DNA binding 1 and JNK signaling	[62]
Platelet-derived	Immune Complex formation	Citrullinated epitopes	[64,70]
Platelet-derived	Pro-coagulatory / platelet activation	P-selectin (?)	[59]
Polymorphonuclear-derived	Pro-inflammatory/ release of IL-6 and IL-8	Endothelial cells	[61]
Monocyte and granulocyte derived	Pro-coagulatory	Thrombin	[59]
FLS-derived	Cartilage damage	MMP-13, MMP-3, IL-6 and VEGF	[61,63]
FLS-derived	Macrophage migration	PTX3 and PSMB5	[12]
B-cell derived	Immune Complex formation	MHC class I and II, CD45RA	[65]
T-cell derived	Acute phase response, complement activation, coagulation (?)	↑DPYSL3 and ↓PSME1	[67]
T-cell derived	Osteoclastogenesis	↓PSME1 / RANKL/RANK	[67]
T-cell derived	Decreased bone repair	↑DPYSL3 / BMP and RhoA	[67]

**Abbreviation:** ↑/↓, up/downregulation.

The pro-inflammatory effect is exerted by leukocyte-derived EVs which have the ability to upregulate the expression of COX-2 and m-PGES-1 in FLSs, promoting thus the release of metalloproteinases through an NF-κB-dependent pathway.^38^ Another postulated role is the promotion of B-cell-activated factor release by FLSs. Platelet-derived EVs, which constitute the majority of EVs in RA, may be implicated in the migration and invasion properties of FLSs mediated by the CXCR2-mediated signaling pathway which leads to the activation of the NF-κB pathway.^40^ Stojanovic et al demonstrated a positive correlation between various EVs in RA, including platelet and monocyte-derived, suggesting their roles in activation coagulation and decreased fibrinolysis.^41^ Moreover, plated-derived EVs are capable of stimulating FLSs through IL-1 receptors which in turn results in the release of IL-6 and IL-8.^42^ These two cytokines are also released by endothelial cells in vitro, in the presence of polymorphonuclear-derived EVs.^43^ Furthermore, endothelial cells are implicated in angiogenesis through an EV-mediated pathway where the encapsulated inhibitor of DNA binding 1 (Id1) activates the JNK signaling.^46^ Other in vitro studies have shown that monocyte and granulocyte-derived vesicles have a pro-coagulatory effect by inducing thrombin formation which suggests a potential connection between inflammation and coagulation, mediated by EVs. Additionally, exosomes derived from FLSs have been shown to contain enzymes for cartilage matrix degradation and to upregulate the expression of MMP-13, MMP-3, IL-6 and VEGF in chondrocytes, further leading to cartilage damage.^70^ Finally, some evidence suggests that platelet-derived EVs carry citrullinated epitopes on their surface, acting as autoantigens which induce immune complex formation.^61^ The same is true for B-cell-derived EVs, which have been shown to carry MHC class I and class II, CD45RA and other molecules responsible for adhesion and immune response activation.^71^ Platelet-derived EVs have been observed to be the most abundant in patients’ plasma and synovium.

A recent study by Varela e*t al* investigates the changes in the dynamic of SF-EVs phospholipidome profile during arthritis, shedding light on a different aspect of EVs’ composition during synovitis. In particular, they propose that specific changes in the lipid contents of EVs are associated with disease and health states. For example, an increase in phosphatidylethanolamines and a sharp upsurge of hexosylceramides were associated with acute inflammation, suggesting a potential role of lipids in synovial inflammation that needs to be further investigated.^72^ In this regard, lipids are known to have both immunomodulatory and anti-inflammatory effects, specifically fatty acids, their metabolites and lipoproteins. Also, specific classes of lipids such as sphingomyelin and hexosylceramides have been associated with EV biogenesis. Examination of those effects is currently at an early stage, and additional research is required to elucidate the role of EV lipid contents in RA.

Another study has demonstrated the potential role of EVs in macrophage migration. Nakamachi et al showed that EVs derived from FLSs in RA do not promote the release of cytokines in macrophages, rather they exhibit a migration effect mediated through PTX3 and PSMB5. The same study confirmed that FLS-EVs contain citrullinated peptides and IgG, suggesting that they also become autoantigens in RA.^12^ Furthermore, a recent proteomic analysis of CD4+ T cells derived EVs revealed the upregulation of DPYSL3 and downregulation of PSME1 in RA patients. This research did not investigate the roles of those proteins; however, authors suggest potential mechanisms in the pathogenesis of RA which include participation, in acute-phase response, complement and coagulation response and PI3K-AKT signaling. In addition, they suggest that downregulation of the proteasome activator complex subunit 1 (PSME1) is implicated in osteoclastogenesis by reducing TNF receptor-associated factor (TRAF6) degradation, an essential protein of signal transduction in the RANK/RANKL signaling pathway. They also suggest, a possible connection between the upregulated DPYSL3 and TNF-α, where DPYSL3 is supposed to affect the repair of damaged bone, through the bone morphogenetic protein (BMP) and *ras* homolog gene family member A (RhoA) signaling.^73^ Some additional new evidence proposes, that the PI3k/Akt pathway might be indeed implicated in the pathogenesis of RA but not EV mediated. Normally, the pathway is responsible for the regulation of the cell cycle. In cancer or hyperplastic tissue, PI3k/Akt is hyperactive, inducing thus proliferation and inhibiting apoptosis. In RA, it is suggested that over-activation of the pathway is a consequence of prolonged inflammation. Researchers connect the over-activation of PI3k/Akt with the overexpression of PRDX4, an antioxidant enzyme, which has been shown to be increased in synovial fluid. In cancer research, where the enzyme has been implicated in bone metastasis, the knock-down of PRDX4 in mice, reduced significantly osteoclast formation, thus becoming a subject of interest in RA as well. Downregulation of PRDX4 resulted in inhibition of proliferation, migration and invasion properties of aggressive FLSs. Upregulation promoted opposite results, respectively. However, PRDX4 is pivotal for the reduction of ROS. Knockdown of PRDX4, results in increased apoptosis due to escalation of intracellular ROS and oxidative stress. Consequently, oxidative stress can result in damage to articular cartilage. Therefore, the role of PRDX4 has not been yet entirely elucidated, especially, since it seems to have a dual effect in RA. In vitro research indicated that silencing of PRDX4, had an inhibitory effect on PI3k/Akt pathway, while the opposite (increased phosphorylation of Akt) has been shown to exert contrasting effects, promoting FLSc proliferation and migration.^74^

Extracellular vesicles developed during inflammation can facilitate intercellular communication through the expression of certain receptors as well. An example is the IL6 signal transducer (IL6ST) also known as Glycoprotein 130 which participates in joint reconstituted signaling (JRS). Interestingly, in this case, there are different modes of action of IL-6 in disease pathophysiology which must be considered when IL-6 inhibitors are administered as form of treatment.^75^ EV receptors might be an interesting area of future exploration since not much research has been published on this matter.

To summarize, EVs depending on their parent cell have distinct but crucial roles in RA pathophysiology, and deregulated paracrine communication mediated by EVs can lead to cartilage damage and bone erosion. Their action is mainly attributed to their contents, eg miRNAs, enzymes, lipids and epitopes and their ability to induce phenotypical changes in target cells. These phenotypical changes translate into pathological conditions in the synovial tissue and can enhance disease progression.

## Outcomes of Current Treatment Methods

Although current therapeutic strategies against RA have been proven to some extent, effective and have decreased morbidity and mortality, many patients still do not respond to administrated treatment or the response is not satisfactory, that is, remission is not achieved. The complexity and heterogeneity of the disease pathophysiology are the main barriers preserving from the development of one universal therapy or cure, however many novel attempts have shown promising in achieving patient remission. While 70–80% of patients respond to treatment and can achieve remission or low disease activity, the remaining, either do not respond to any kind of treatment since diagnosis or their response decreases over time due to immunogenicity, for example, TNF-α inhibitor failure. This type of failure is associated with a high presence of anti-drug antibodies (ADA), which have been correlated with treatment impairment.^76^ Another example of an ineffective response mechanism is the low frequency of Tregs during RA, which has been associated with decreased tolerance to DMARDs. Differentiation of Tregs, however, is regulated by IL-6, and inhibition of IL-6 with targeted biological therapy such as Tocilizumab can restore Tregs frequency, thus increasing DMARDs tolerance.^77^ Inadequate response to treatment can also be related to genetic polymorphisms. In particular, it has been demonstrated that polymorphisms within genes encoding for RANK/RANKL, might be related to disease susceptibility and treatment outcomes when TNF-α inhibitors are administered.^78^ Similarly, polymorphisms within genes encoding for TNF-α and TNF receptor, specifically homozygosity of the TNFR1A 36A allele and the TNFA-875T variant, might be implicated in therapy response.^79^ Studies have also examined the polymorphisms of IL-6, rs1800795 and rs12083537 and suggesting their associations to biological DMARDs response.^80^,^81^

Although targeted treatment with biological DMARDs, is an effective alternative when first-line treatment or administration of synthetics and/or glucocorticoids fail, the cost of treatment prevents many from accessing different types of medication, especially for prolonged periods of time. Furthermore, although generally considered safe for use, the available treatments may produce a series of side effects such as bone marrow toxicity, increased risk of infections, gastroenterological injury, renal failure, and dermatological, cardiovascular and hematological issues.^7^,^82^ The drug pharmacokinetics must also be considered since in certain cases drugs are unable to pass effectively through the system, directly into the inflamed joints which naturally are not rich in the vasculature, resulting thus in poor absorption and consequently insufficient patient response.^83^,^84^

There is no doubt that to improve personalized medicinal care of RA patients, especially those who do not respond to any type of treatment or who develop resistance, new therapies are essential.

## Therapeutic and Diagnostic Potential of EVs

EVs have been proposed and tested as therapeutic agents and biomarkers in various diseases, especially cancer and autoimmune disorders. Their potential in RA has been explored in vivo and in vitro from various perspectives, including genetic modifications of carried miRNAs, drug delivery, immunoregulation and/or immunosuppressive action or cargo interference by loading specific molecules. The advantages of EVs in therapeutics are their excellent biocompatibility, lack of toxicity, inability to proliferate (avoiding thus tumor formation) and functional and structural stability.^85–88^

Mesenchymal stem cells (MSCs) are stromal cells that have the ability to self-renewal and multilineage differentiation which leads to the formation of mesenchymal tissue, including cartilage and bone, while they are also known for their immunomodulatory roles.^89^ The same properties are observed in MSC-derived EVs which could be useful in terms of cartilage repair, decrease in bone destruction and immunoregulation in RA. Promising in clinical application seems to be the use of human umbilical cord mesenchymal stem cell-derived exosomes (ucMSC-EVs) loaded with miR-451a, which were shown to modulate the expression ATF2 in aggressive phenotype FLSs, thus inhibiting their proliferation, migration and invasion both in vivo and in vitro. Improvement in inflammation and disease manifestation was observed in mice.^9^ Previous study of miRNA MSC-derived exosome content, specifically miR-150, identified that these exosomes were able to interfere with the FLSs aggressive phenotype, by targeting MMP14 and VEGF in mice. Reduced angiogenesis and hyperplasia in the synovium were noted as well.^90^ MSC-derived EVs have been described as paracrine messengers and have also been investigated in terms of immunosuppression. Interestingly, they have been shown to inhibit T-cell proliferation and decrease CD4+ and CD8+ while increasing the presence of T regs in collagen-induced arthritis (CIA) in vivo. The same study highlighted the advantages of EVs in comparison with other microparticles, with EVs, having more prominent immunomodulatory roles.^91^

One interesting approach for EVs implementation in RA treatment has been proposed by Kim et al, who focused on macrophage-derived EVs for in situ reprogramming. Specifically, they performed in vivo conversion of pro-inflammatory M1 into reprogrammed anti-inflammatory M2 through M2-derived EVs, thus achieving an equilibrium between M1 and M2. This equilibrium is disrupted in RA synovial tissue and hence it is difficult to achieve remission results by macrophage depletion, which can have adverse effects such as infections. Repolarization of macrophages through incubation of M2-EVs with active M1 macrophages resulted in a decrease of inflammation and anti-arthritic effect in mice, without severe complications. This effect was attributed to the expression of specific proteins, such as MMP-19, CCL8, glutamine synthetase and MFG-E8 by M2-EVs.^11^ Repolarization of macrophages has been also investigated through exosomes from adipose-derived stem cells, which were shown to regulate macrophage phenotype and bone tissue healing in vivo, partly by miR-145a. This study was performed in rats, however, not with CIA. Nonetheless, the results in accordance with the results of Mi et al suggest that further research on exosomes and their miR-145a content in the context of RA might be interesting.^92^

Drug loading of exosomes has also been attempted in terms of RA. In their study, Li e*t al*, produced engineered M2-derived exosomes loaded with curcumin penetrating peptide modification (Cur@EXs-R9). In mice, potent accumulation of exosomes in inflamed sites decreased inflammation and improved motor function were observed, while in vitro, repolarization of macrophages occurred.^93^ A different approach to drug-loading was tested by Rao e*t al* who encapsulated triptolide in dendritic cells-derived exosomes. Triptolide has been shown to be effective in the treatment of autoimmune diseases, although it exerted high toxicity. When loaded into EVs and administered to mice, no toxicity was observed. Additionally, this treatment-induced T-cell differentiation in vitro, a decrease of CD4+ and an increase in the presence of T regs in vivo.^94^

The biomarker potential of circulating and synovial fluid EVs has also been characterized with focus on miRNAs.^95^,^96^ Possible biomarkers could be exosomal miR-335-5p and miR-486-5p, whose expression has been higher in RA patients compared to controls. Positive correlations were found between hsa-miR-335-5p and antiCCP, DAS28-ESR, DAS28-CRP, and RF which are standard biomarkers. A common target of these exosomal miRNAs, the *SRSF4* has marked a decrease in synovial fluid, suggesting that along with miR-335-5p and miR-483-5p, they could be promising disease biomarkers.^10^ Other exosomal biomarkers, found in the serum of RA patients of a pilot study, were the exomiR-451a and exomiR-25-3p which along with the sTWEAK protein, correctly classified 95.6% of patients in the early disease stage. Tumor necrosis factor-like weak inducer of apoptosis (TWEAK) is an inflammatory cytokine involved in the pathogenesis of RA, specifically FLSs proliferation, bone erosion and cytokine production, through signal transducer receptor Fn14 binding. sTWEAK is the TWEAK soluble form. The exosome-derived miRNAs, were elevated, while overall 11 differentially expressed exosomal miRNAs were identified in the same study.^97^ In earlier research, 20 exosomal miRNA were differentially expressed, with an additional biomarker identified, the miR-548a-3p. In this research, low levels of this particular sequence were associated with higher disease activity and the TLR4/NF-κB signaling pathway.^98^ Exosomal miRNA biomarkers have the advantage of specificity and possible detection at the early disease stage, which is often not the case with standard biomarkers such as ACPAs where false positives can arise or when patients have seronegative phenotype.

## Challenges with EV Treatment and Future Perspectives

Research so far indicates that extracellular vesicles, with focus on exosomes, can become a promising alternative for the treatment and diagnosis of RA. Nevertheless, some considerations and challenges regarding their implementation in clinical practice must be addressed. First, due to their heterogeneity and small size, exosomes are difficult to separate and isolate. It is also a challenge to differentiate between exosomes and other types of EVs. There are no standardized methods for this process, and different techniques or variations are seen within literature. A collective standardized method or combination of methods with adequate specificity and sensitivity will be necessary for clinical practice in order to obtain pure and high-quality exosomes for diagnosis and treatment. Although exosomes are generated by the host organism with minimal chances of rejection or immediate harm, their administration as drug delivery vesicles or genetic modifiers must be tested in terms of short and long-term safety and dosing, which is yet to be performed. When considering clinical trials and then massive treatment, the issue of EV production and engineering arises. Not only the cost but also the fact that EVs are not naturally produced in high amounts will be challenging to overcome. Clearance of EVs from the host will be another aspect to address before clinical application. As there are no standardized separation methods, there are also no standardized drug loading or manipulation methods, and the research in this area is still a pioneer. Separation, manipulation and loading procedures can affect the quality, purity and functionality of the vesicles. The choice of the appropriate exosomes is another aspect to be considered. It must be noted that exosomes often contain information about their parent cells but that is not always the case. When choosing specific exosomes derived from parent cells, their functional characteristics and rate of release must be known. What remains elusive is the molecular mechanism of EV pathogenesis in RA, where additional research is required for a better understanding of EVs’ behavior. Particularly, since EVs could be used as potential therapeutic agents, it is important to be able to distinguish between those which are related to disease development and progression and those which are administered for treatment purposes. As it has been demonstrated, treating RA, using EVs, might become a promising method with various advantages. Large-scale studies are encouraged to address the current limitations and make use of EVs’ potential in therapeutics.

## Conclusion

The pathogenesis of rheumatoid arthritis, is a complex and multifactorial concept, which is not yet completely understood. For this reason, a certain percentage of patients do not respond to available treatments or their response is not satisfactory. Genetic predispositions and epigenetic changes contribute to disease development and heterogeneity. One interesting contributor to disease pathophysiology might be extracellular vesicles, specifically exosomes, whose role seems to be crucial in intercellular communication and immunomodulation in RA. In vivo and in vitro studies demonstrate a potential for RA treatment with the use of exosomes, however many challenges and limitations prevent their implementation. Understanding RA pathophysiology and, more specifically, the implication of EVs and exosomes can open a new perspective for RA treatment.
